# Software for assessment and monitoring the decoding skills development of children from the elementary school: validity based on response process

**DOI:** 10.1590/2317-1782/20242023349en

**Published:** 2024-09-13

**Authors:** Aparecido José Couto Soares, Débora Maria Befi-Lopes

**Affiliations:** 1 Departamento de Fonoaudiologia, Escola Paulista de Medicina – EPM, Universidade Federal de São Paulo – UNIFESP – São Paulo (SP), Brasil.; 2 Departamento de Fisioterapia, Fonoaudiologia e Terapia Ocupacional, Faculdade de Medicina, Universidade de São Paulo – USP – São Paulo (SP), Brasil.

**Keywords:** Reading, Learning, Child, Teaching, Software

## Abstract

**Purpose:**

To continue the validation process of the Decoding Development Monitoring Protocol (PRADE) in software format in the validity evidence stage based on response processes.

**Methods:**

250 individuals participated in this study, 125 individuals from private schools and 125 individuals from public schools. The assessment was carried out in person using the software that hosts the instrument's tasks, which are organized into decoding linguistically balanced words and non-words, respecting the decoding rules of Brazilian Portuguese. The software prepares an individual performance report for each participant, counting the decoding time for each stimulus, as well as the number of words decoded correctly. The data is organized considering the correct decoding time of the stimuli, decoding accuracy and percentage of correct answers. All data underwent statistical analysis using SPSS software.

**Results:**

The data indicated an important effect of the length of words and non-words on public and private school students. Furthermore, it was possible to observe the evolution of decoding, depending on the school year, in all the variables studied. In both groups, a strong influence of non-words on student performance throughout Elementary School I was observed.

**Conclusion:**

The data indicate validity in the analysis of response processes, since it was possible to adequately characterize the performance of school children public and private throughout Elementary School I, characterizing each group, as well as their differences according to the advancement of schooling.

## INTRODUCTION

The assessment of decoding fluency through read-aloud practice is the most common method to monitor children's acquisition and progress, both regarding school evaluation reports as well as to verify the effectiveness of the stimulation and/or intervention programs^(1,2)^. In fact, oral decoding assessment results are so important that they are considered to be fundamental predictors of the individual's reading performance. In the United States, for instance, oral reading fluency assessment scores are analyzed by the Federal Education Department to monitor the children's academic development and for the improvement of early stimulation programs^(1,2)^.

Oral reading of isolated words is undoubtedly the most widely used task to assess an individual's decoding proficiency. This is partially due to the fact that this specific task isolates the contextual or visual (pictorial) cues, therefore, the evaluation is strictly restrained to the decoding skill^(2)^. Reading assessment instruments are strongly based on the dual-route model, since they use words and non-words^(2)^. It is important to emphasize that efforts have been made to create and validate reading tests for Brazilian Portuguese (BP) speakers^(3-6)^, however, most of them are more focused on reading and its processes instead of isolated decoding, not to mention the insufficient investigation on the tests’ psychometric properties^(7-9)^.

According to the literature^(10)^, children who present any delay or fail to acquire the automatic word recognition skill may be at risk for several alterations in written language learning. Nevertheless, despite the advances in relation to the study of reading regarding its parameters and underlying skills, the number of validated instruments for an effective evaluation and intervention of potential reading and learning disorders is still reduced in Brazil^(3-9)^. That being said, most of these instruments are not in a software or application format, which not only brings the decoding assessment process into the twenty-first century, but can also provide a more reliable data concerning the decoding time, the number/percentage of correct answers or accuracy, and the number of words read correctly per minute or in one minute, which are all indicators reckoned as the gold standard in international decoding tests. The EduEdu® application is an exception in this case, providing activities that allow to evaluate and monitor the learning of the written language in a holistic approach, despite not having the appropriate scientific validation^(11)^.

In a country with an educational reality such as Brazil’s, in which it is recurrent for students to experience some degree of difficulty reading or writing, it is essential to characterize the children’s reading skill earlier on, especially in the learning phase of decoding. This takes into consideration the latest report of the Programme for International Student Assessment – PISA, in which Brazil continues to exhibit high rates of school failure^(12)^. According to published data, the country has remained among the worst-performing countries for more than a decade. Furthermore, the abovementioned report indicates that Brazilian schoolchildren’s reading difficulties usually onset in basic education, that is, related to the decoding competence, interfering with the consolidation of literacy. When misidentified, these difficulties can become chronic, triggering a low performance throughout the student’s school career^(12)^.

As previously stated, the lack of properly validated instruments prevents professionals that are directly involved in working with schoolchildren (speech language therapists, teachers and psychologists) from establishing adequate parameters to identify the decoding difficulties and alterations, consequently hindering the development of more effective stimulation strategies, including in the classroom^(10)^. This reality is quite different in developed countries, where there is a standard use of validated tests to assess decoding, fluency and reading comprehension. Conventionally, these instruments are composed of a list of words and texts that must be read by the children at a predetermined interval of time^(10,13,14)^.

Hence, the elaboration of a Brazilian decoding assessment instrument that could be used as a tool to monitor this skill’s development is fundamental for both the clinical and the educational fields. Moreover, it is extremely necessary to have such an instrument in a software format*,* in which performance can be measured more reliably, being suitably validated with the psychometric characteristics analyzed and with free access for speech language therapists, educational psychologists, parents, teachers or further professionals who work in learning. This way is possible to build a broad support network and supervise the decoding development, which is characterized by a multidisciplinary approach along with the construction of stronger relationships between professionals and the child's family^(15)^. It is crucial to highlight that monitoring instruments should be applied to healthy as well as to unhealthy individuals, targeting the identification of those presenting risk factors for certain conditions or disabilities, so that the early identification can produce better results^(16)^. To be effective, the instrument must be validated, be straightforward and have a simple application, reproducibility and accuracy^(16)^.

Consequently, the objective of the present study is to proceed with the validation process of the Decoding Development Monitoring Protocol (*Protocolo de Acompanhamento do Desenvolvimento da Decodificação* - PRADE), in a software format*,* regarding the validity evidence based on the response processes, since all previous steps have already been published and referenced ^(17-19)^.

## METHOD

This research was approved by the Research Ethics Committee of the University of São Paulo Medical School (*Faculdade de Medicina da Universidade de São Paulo* - FMUSP) (REC No. 2,262,300). This is a prospective study conducted in accordance with the principles of the Standards for Educational and Psychological Testing (SEPT)^(20)^, a guideline proposed by American organizations that compiles recommendations and fundamental definitions regarding the psychometric aspects involved from the preparation to the interpretation of the tests, accompanied by the different steps necessary to validate an instrument. The data collection procedures herein only began after the signing of the Informed Consent Form both by the school involved in the study and by the parents/guardians, in addition to the signing of the Consent Form by the children.

### Case study

250 individuals participated in this study, 125 individuals from private schools and 125 individuals from public schools. Both groups were subdivided into five equal sized (No. = 25) groups according to the school year, that is, from the first to the fifth school year. To be included in the present study, children were required to meet the following criteria: be regularly enrolled in Elementary School; absence of complaints/ indicators of hearing or visual alterations; absence of indications of neurological or cognitive disorders; absence of retention in the school record; absence of phonological and oral language alterations, as verified through speech-language screening.

In order to ensure that the study sample comprised children with different academic profiles and to avoid singling out only one profile of children screened for participation, whether those with better academic performance or those with a greater difficulty to learn decoding, it was decided to use a stratified random sampling for the participant selection. Therefore, the children were initially numbered from 1 to 150, in ascending order according to the school year, in both schools. Then, these numbers were used to randomly select the final study sample, as characterized below.

As a result, the public school group was composed as follows: 25 1st grade children (12 girls and 13 boys; mean age of 6.56); 25 2nd grade children (14 girls and 11 boys; mean age of 7.47); 25 3rd grade children (11 girls and 14 boys; mean age of 8.65); 25 4th grade children (12 girls and 13 boys; mean age of 9.64); 25 5th grade children (10 girls and 15 boys; mean age of 10.52 s). The private school group was constituted as follows: 25 1st grade children (14 boys and 11 girls; mean age of 6.60); 25 2nd grade children (12 girls and 14 boys; mean age of 7.25); 25 3rd grade children (11 girls and 14 boys; mean age of 8.52); 25 4th grade children (14 girls and 11 boys; mean age of 9.56); and 25 5th grade children (11 girls and 14 boys; mean age of 10.52).

#### Process

Considering that the present study is part of the PRADE validation process, it is important to emphasize that the following steps of validity evidence based on the test contents; delimitation of the target population; elaboration of the items; analysis of judges with expertise in the area; determination of the sample size; protocols to verify that the population understands the test items; application of the test in a sample of the target population; data analysis and correlations were carried out in a previous study and published in an international journal^(17)^ confirming the validity of these procedures.

Under these circumstances, it was possible to proceed to the stage of validity evidence based on the response processes, which is characterized by the assessment of different strata of the target population’s performance in the task application, seeking to understand the processes involved in the response patterns^(21)^. It is worth noting that phases of this stage have already been performed in previous studies with children presenting Developmental Language Disorder (DLD), which analyzed this population’s performance in the test presented herein, as reported in two studies published in journals indexed within the Web of Science with a relevant impact factor^(18,19)^. The data suggested a difference in the performance of subjects with DLD when compared to their neurotypical peers, indicating an important discriminant validity of this instrument regarding neurotypical children and those with DLD. However, it is necessary to confirm this last aspect in future studies with different populations.

It should also be pointed out that, when it comes to preparing the materials to verify aspects of written language in Brazil, there is a history of poor performance in both national and international assessments, along with the literacy rates and alarming functional illiteracy that should also be taken into account, especially pertaining students from public schools and those in the intersectionality of public school added to social vulnerability. In this sense, it is valid to further verify the effectiveness of this instrument to adequately characterize the performance of public and private school students.

PRADE consists of linguistically balanced words elaborated according to the decoding rules of BP^(22)^, respecting the word length variation from mono to polysyllables appropriate to children in this school level^(23)^. Furthermore, the test has non-words that were derived from real words, likewise following the BP decoding rules and the variation from mono to polysyllables^(23)^.

**Figure 1:**
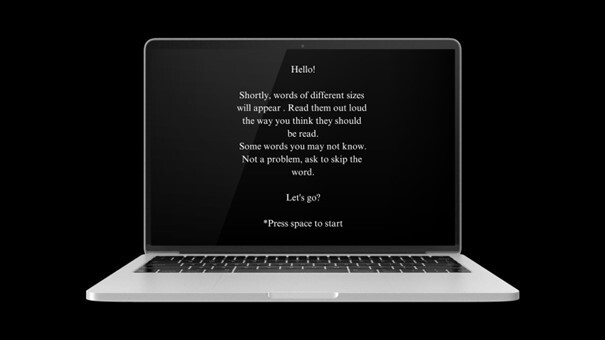
PRADE home screen displaying the welcome message and instructions

The assessment was performed in person using the software that hosts PRADE's tasks, Psychopy®, on the participants' extracurricular time. It is noteworthy that the data collection occurred in the second semester of the school year, as this ensures that the academic skill profile is consistent with the individuals’ school year, especially with regard to students in the first school year. When starting the software, a home screen appears welcoming the participant and explaining how the test works (Figure 1).

**Figure 2:**
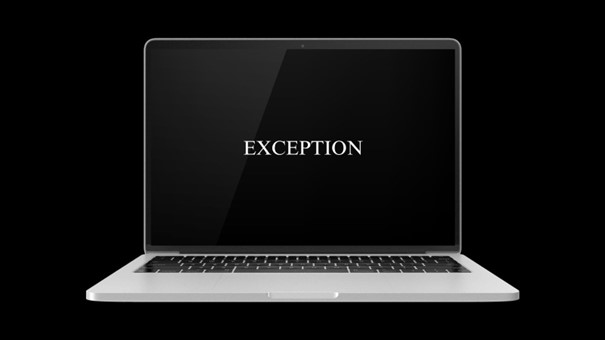
Example of how stimuli appear in PRADE

The task begins with real words that are randomly presented in arial font, uppercase, No. 20 written in white on a screen with a gray background (Figure 2).

**Figure 3:**
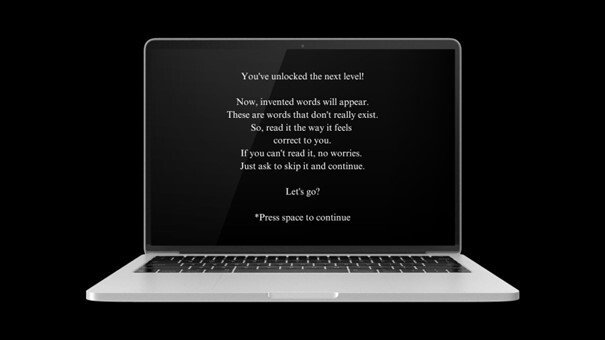
Non-word decoding start screen

During the assessment, the evaluator presses the number 0 for each wrong decoding response and the number 1 for each correct decoding. It should be made clear that “correct decoding” was considered to be the response consistent with the grapheme-phoneme correspondences, spelling rules and adequate tonicity in stimuli with diacritical marks^(22)^. Subsequently, the decoding of the non-words starts by showing the participant a new screen presenting the new instructions (Figure 3).

The procedure for evaluating the decoding of non-words is analogous to the one described for word decoding. It must be stressed that, for the characterization of the non-words’ correct or incorrect decoding, it was strictly considered the participant's ability to use BP decoding rules, including those that contained some diacritical mark^(22)^. Specifically, the evaluator read the on-screen instructions to the participants to ensure that the task was fully understood. Ultimately, each child took an average of 2 minutes and 30 seconds to complete the entire task.

The software used herein produces an individual performance report for each participant, accounting for the decoding time of each stimulus, from monosyllables to polysyllables, along with the number correctly decoded words. Next, the data is tabulated considering the correct word decoding time, the percentage of correct answers per stimulus length (from mono to polysyllable) and their total scores, as well as the decoding accuracy, namely, the number of words/non-words correctly decoded per minute. The stimulus length and its total scores were also considered, for both words and non-words. It is essential to highlight that, in the analysis of the correct words and non-words decoding time, participants who did not correctly decode any stimulus were excluded. All data were statistically analyzed using the SPSS Statistics software, version 28.0 (IBM Corp., Armonk, NY, USA).

## RESULTS

Table 1 depicts the central tendency and dispersion measures of the decoding time for each type of school according to the school year, word length and stimulus type; word/non-word. The data indicate a greater number of children in the first year of private schools who were able to effectively decode words and non-words, the number decreases as for public school participants depending on the stimulus size. The influence of the stimulus length is evident in further school years, showing a closer approximation of public and private school participants’ decoding time, as well as an increase in the number of children who were able to perform the task from the second year onwards. It was demonstrated that, following the course of the ensuing schooling process, both the number of participants and the proximity of the correct decoding times reaches an equivalence point, stabilizing in all participants from the third school year onwards.

**Table 1:** Descriptive scores and comparative analysis of school types in relation to the correct word reading time according to word length, school and word types

**Variables**		**Words**								**Non-Words**					
**School Year**	**Length**	**School Type**	**No.**	**Average**	**SD**	**Median**	**Min.**	**Max.**		**No.**	**Average**	**SD**	**Median**	**Min.**	**Max.**
1	Mono	Public	14	6.16	3.01	7.04	1.40	12.01		6	9.65	2.87	8.65	6.50	14.60
		Private	23	9.28	8.49	7.00	.90	45.80		22	10.39	3.95	9.95	2.10	19.50
	Di	Public	14	28.34	16.01	32.75	1.10	51.30		5	27.64	3.34	28.20	22.20	31.20
		Private	20	20.12	7.60	20.20	3.10	36.20		20	40.88	11.36	39.80	21.20	59.10
	Tri	Public	13	17.20	14.91	12.10	5.50	55.52		8	38.84	23.75	49.20	7.60	68.20
		Private	20	31.04	9.81	34.20	10.10	45.30		20	64.98	27.32	63.95	33.40	142.90
	Poli4	Public	5	21.60	10.05	25.30	5.20	30.10		5	30.28	17.88	22.20	12.40	55.30
		Private	19	72.51	32.62	67.10	23.40	137.40		19	50.79	24.66	52.30	8.20	98.20
	Poli5	Public	4	26.63	8.32	29.90	14.50	32.20		4	26.08	14.25	26.70	8.20	42.70
		Private	19	30.86	14.43	32.10	8.20	66.00		17	28.18	13.25	25.70	8.30	49.60
	Total	Public	15	61.41	47.66	49.70	4.60	161.91		9	84.72	75.60	103.90	6.50	192.80
		Private	23	145.43	60.79	157.85	6.10	262.40		22	172.27	74.47	171.00	3.20	289.80
2	Mono	Public	23	9.24	3.55	9.20	1.20	16.50		23	10.10	2.87	10.10	4.50	17.20
		Private	25	7.96	1.87	7.50	5.10	14.90		24	8.66	1.64	8.50	4.20	11.60
	Di	Public	24	28.03	10.76	28.90	7.50	57.90		22	31.19	9.75	32.85	4.10	54.50
		Private	24	24.65	5.44	24.65	15.40	38.20		24	30.12	7.81	29.60	19.80	48.20
	Tri	Public	21	40.99	11.41	41.20	22.20	68.90		21	45.25	13.51	44.60	19.20	86.70
		Private	24	36.89	10.52	34.70	19.20	59.20		24	43.35	9.12	41.75	22.30	59.20
	Poli4	Public	21	38.70	9.76	39.80	14.90	64.50		21	39.43	13.76	38.90	12.20	61.20
		Private	24	35.88	8.84	35.70	21.70	55.30		24	33.66	11.86	36.35	5.10	53.20
	Poli5	Public	21	31.72	8.65	32.30	9.20	48.20		20	21.67	7.71	21.20	6.70	35.70
		Private	24	25.03	9.30	24.05	5.70	54.20		22	21.95	11.72	22.40	6.30	43.80
	Total	Public	24	134.37	52.42	147.25	8.20	208.80		23	136.08	51.19	149.10	8.60	221.80
		Private	25	125.51	35.25	131.00	14.90	177.70		24	135.91	32.63	140.40	70.80	192.90
3	Mono	Public	24	7.66	1.41	7.55	4.70	11.30		25	8.03	2.09	8.80	2.40	11.70
		Private	25	7.11	1.45	6.70	5.50	12.40		25	7.89	2.20	7.80	3.90	14.70
	Di	Public	24	24.56	5.78	23.10	18.70	44.90		24	28.65	4.81	28.05	19.20	39.10
		Private	25	20.58	4.09	19.20	14.10	32.10		25	28.56	8.78	26.50	19.50	54.20
	Tri	Public	24	40.81	7.14	39.50	27.40	56.70		24	47.39	7.88	45.95	34.20	61.20
		Private	25	36.25	10.84	34.10	20.80	62.20		25	40.26	9.63	37.20	28.20	57.20
	Poli4	Public	24	37.18	8.58	37.20	22.20	57.20		24	63.16	104.56	40.85	22.10	550.70
		Private	25	33.03	10.07	29.80	18.10	55.40		25	28.26	8.30	29.10	12.30	49.50
	Poli5	Public	24	22.66	4.30	22.70	15.30	32.20		23	25.53	10.86	24.20	6.20	55.20
		Private	25	19.33	5.52	18.60	12.10	31.20		25	20.47	8.14	19.60	6.10	33.90
	Total	Public	24	132.87	23.21	133.10	97.90	200.20		24	171.93	103.19	155.30	92.50	639.70
		Private	25	116.31	25.06	115.50	85.50	171.20		25	125.44	26.31	127.10	78.10	176.40
4	Mono	Public	25	7.06	1.38	7.10	5.10	11.20		25	7.38	1.83	6.90	4.50	11.70
		Private	25	7.12	1.06	7.00	5.10	8.90		25	7.94	1.83	7.60	5.00	13.20
	Di	Public	25	22.29	4.63	21.20	16.20	39.20		25	23.48	4.96	22.40	14.50	37.50
		Private	25	21.13	2.87	20.70	17.20	29.20		25	26.76	6.70	26.40	17.40	52.80
	Tri	Public	25	38.56	8.14	38.10	24.70	52.90		25	36.08	8.99	33.40	22.30	60.70
		Private	25	37.06	9.96	34.70	26.40	66.20		25	35.77	8.64	34.50	23.20	53.20
	Poli4	Public	25	34.51	7.91	32.60	22.00	50.10		25	37.86	8,43	41.20	12.30	48.20
		Private	25	31.50	9.16	30.00	19.90	63.30		25	35.86	10.50	32.20	22.40	59.10
	Poli5	Public	25	20.27	6.45	20.70	12.30	38.20		25	24.52	9.18	24.50	10.10	41.20
		Private	25	18.54	5.80	16.50	13.20	37.10		25	22.86	6.78	22.20	14.50	45.20
	Total	Public	25	122.68	21.03	121.30	83.30	167.30		25	129.30	23.20	130.90	84.90	187.40
		Private	25	115.35	27.26	105.90	82.30	204.70		25	129.19	24.83	124.50	93.60	181.80
5	Mono	Public	25	7.74	1.18	7.70	5.70	10.30		25	8.60	3.11	8.30	4.10	19.20
		Private	25	7.56	1.95	7.30	4.70	14.70		25	8.59	2.54	7.80	4.70	15.60
	Di	Public	25	23.11	4.23	23.00	17.90	34.10		25	25.98	6.03	25.90	15.20	40.20
		Private	25	22.28	3.63	21.10	17.10	30.20		25	25.82	4.80	25.00	18.00	38.10
	Tri	Public	25	36.08	7.47	35.40	20.80	53.20		25	40.31	10.42	41.20	22.20	55.20
		Private	25	34.88	7.98	33.20	21.20	54.60		25	39.32	9.35	38.20	23.20	61.20
	Poli4	Public	25	32.69	7.94	30.50	22.00	50.10		25	38.90	10.07	39.10	23.40	63.20
		Private	25	31.24	8.11	29.10	21.70	53.40		25	35.84	12.93	34.20	12.20	79.20
	Poli5	Public	25	19.77	4.97	18.70	12.00	28.60		25	23.91	7.51	22.30	8.10	46.10
		Private	25	17.37	4.72	16.40	10.70	29.30		25	23.78	8.66	21.10	6.20	49.10
	Total	Public	25	119.39	21.50	114.10	88.50	168.80		25	137.70	24.49	133.60	99.80	193.40
		Private	25	113.34	20.49	108.40	86.80	160.80		25	133.35	27.59	132.40	90.00	209.40

**Caption:** SD: Standard deviation; Min.: Minimum; Max: Maximum

Table 2 presents the central tendency and dispersion measures related to the correct answer percentage for each school type according to the school year, word length and of stimulus type; word/non-word. Once again, the data corroborates the influence of the stimulus length on the correct answer percentage for children from both schools, through all school years, especially concerning non-words. It is notable that private school students exhibit a higher correct answer percentage through all school years and for all stimuli lengths. The data also indicate the similarity of the students' performance from the third school year onwards, indicating a performance stabilization in later school years.

**Table 2:** Descriptive scores and comparative analysis of school types in relation to the correct answer percentage according to word length, school year and word type

**Variables**				**Words**						**Non-Words**					
**School Year**	**Length**	**School Type**	**No.**	**Average**	**SD**	**Median**	**Min.**	**Max.**		**No.**	**Average**	**SD**	**Median**	**Min.**	**Max.**
1	Mono	Public	25	23.33	34.36	16.67	0.00	100		25	15.33	29.23	0.00	0.00	83.33
		Private	25	74.00	35.38	83.33	0.00	100		25	62.00	32.46	66.67	0.00	100
	Di	Public	25	15.75	26.34	6.25	0.00	87.50		25	11.50	24.98	0.00	0.00	75.00
		Private	25	57.00	34.63	68.75	0.00	93.75		25	58.25	35.07	75.00	0.00	93.75
	Tri	Public	25	12.18	22.87	4.55	0.00	86.36		25	10.91	21.80	0.00	0.00	68.18
		Private	25	43.82	29.45	50.00	0.00	86.36		25	45.64	28.85	54.55	0.00	86.36
	Poli4	Public	25	8.00	19.97	.00	0.00	81.25		25	5.50	12.67	0.00	0.00	43.75
		Private	25	46.00	33.07	56.25	0.00	93.75		25	36.75	29.50	37.50	0.00	87.50
	Poli5	Public	25	10.50	26.19	.00	0.00	100		25	6.50	16.97	0.00	0.00	62.50
		Private	25	48.00	34.74	50.00	0.00	100		25	32.50	31.87	37.50	0.00	100
	Total	Public	25	12.46	23.20	4.29	0.00	85.71		25	9.37	19.49	0.00	0.00	61.43
		Private	25	49.09	30.62	62.86	0.00	90.00		25	45.09	28.23	57.14	0.00	81.43
2	Mono	Public	25	72.00	30.32	83.33	0.00	100		25	60.00	27.22	66.67	0.00	100
		Private	25	96.67	9.62	100	66.67	100		25	79.33	22.71	83.33	0.00	100
	Di	Public	25	62.50	28.30	62.50	0.00	100		25	62.75	32.05	68.75	0.00	100
		Private	25	76.50	21.29	87.50	0.00	93.75		25	76.75	24.44	81.25	0.00	100
	Tri	Public	25	51.09	29.32	54.55	0.00	95.45		25	43.27	25.62	45.45	0.00	86.36
		Private	25	68.36	24.81	77.27	0.00	100		25	58.00	19.21	63.64	0.00	81.82
	Poli4	Public	25	49.25	29.50	50.00	0.00	93.75		25	35.75	23.21	37.50	0.00	87.50
		Private	25	69.75	24.18	75.00	0.00	100		25	42.75	21.70	43.75	0.00	75.00
	Poli5	Public	25	56.50	31.69	62.50	0.00	100		25	36.00	26.59	37.50	0.00	100
		Private	25	70.50	27.21	75.00	0.00	100		25	40.50	28.48	37.50	0.00	87.50
	Total	Public	25	54.23	27.42	57.14	0.00	91.43		25	45.37	24.85	48.57	0.00	88.57
		Private	25	71.26	20.60	74.29	5.71	92.86		25	56.97	19.74	60.00	0.00	81.43
3	Mono	Public	25	92.00	21.04	100	0.00	100		25	77.33	18.56	83.33	16.67	100
		Private	25	98.00	5.53	100	83.33	100		25	82.00	17.29	83.33	50.00	100
	Di	Public	25	86.50	20.06	87.50	0.00	100		25	83.25	20.23	87.50	0.00	100
		Private	25	89.50	6,68	87.50	81.25	100		25	87.50	11.69	87.50	50.00	100
	Tri	Public	25	81.45	19.28	81.82	0.00	100		25	68.36	19.48	68.18	0.00	90.91
		Private	25	92.18	8.85	95.45	54.55	100		25	66.73	11.86	68.18	31.82	86.36
	Poli4	Public	25	82.75	20.19	87.50	0.00	100		25	59.50	16.74	62.50	0.00	81.25
		Private	25	89.00	9.76	87.50	62.50	100		25	56.75	18.92	62.50	12.50	87.50
	Poli5	Public	25	81.50	20.45	87.50	0.00	100		25	55.00	28.18	62.50	0.00	100
		Private	25	89.00	15.44	100	50.00	100		25	60.50	24.12	62.50	12.50	87.50
	Total	Public	25	81.49	18.55	85.71	0.00	95.71		25	66.97	17.43	70.00	0.00	85.71
		Private	25	88.34	6.26	90.00	65.71	95.71		25	67.89	12.17	70.00	37.14	82.86
4	Mono	Public	25	93.33	8.33	100	83.33	100		25	72.67	17.27	83.33	33.33	100
		Private	25	96.67	8.33	100	66.67	100		25	85.33	13.88	83.33	66.67	100
	Di	Public	25	88.75	8.84	93.75	62.50	100		25	72.75	18.39	75.00	31.25	100
		Private	25	91.75	7.59	93.75	75.00	100		25	90.75	11.71	93.75	50.00	100
	Tri	Public	25	87.82	11.49	90.91	59.09	100		25	61.27	13.89	63.64	27.27	81.82
		Private	25	96.73	5.33	100	77.27	100		25	74.36	12.71	72.73	45.45	100
	Poli4	Public	25	83.00	16.19	87.50	37.50	100		25	56.50	14.93	62.50	12.50	81.25
		Private	25	96.75	4.46	100	87.50	100		25	65.25	15.84	68.75	43.75	100
	Poli5	Public	25	88.50	12.97	87.50	50.00	100		25	60.00	23.11	62.50	12.50	87.50
		Private	25	98.50	4.15	100	87.50	100		25	71.00	16.82	75.00	37.50	100
	Total	Public	25	84.97	9.04	87.14	60.00	95.71		25	61.89	13.48	65.71	22.86	77.14
		Private	25	93.03	3.72	94.29	81.43	97.14		25	74.46	9.65	75.71	52.86	91.43
5	Mono	Public	25	92.67	9.72	100	66.67	100		25	78.67	15.61	83.33	50.00	100
		Private	25	96.67	6.80	100	83.33	100		25	84.67	14.37	83.33	50.00	100
	Di	Public	25	91.00	6.01	93.75	75.00	100		25	76.75	14.38	75.00	43.75	100
		Private	25	93.25	5.68	93.75	81.25	100		25	88.75	11.69	93.75	56.25	100
	Tri	Public	25	92.73	9.00	95.45	63.64	100		25	69.64	14.05	72.73	22.73	95.45
		Private	25	94.18	10.21	100	54.55	100		25	72.55	13.67	72.73	45.45	100
	Poli4	Public	25	92.00	8.75	93.75	62.50	100		25	65.50	17.60	68.75	31.25	87.50
		Private	25	93.75	7.22	93.75	75.00	100		25	63.50	17.09	68.75	18.75	87.50
	Poli5	Public	25	94.00	9.63	100	62.50	100		25	63.00	24.60	62.50	12.50	100
		Private	25	94.00	8.93	100	75.00	100		25	68.00	24.49	75.00	12.50	100
	Total	Public	25	89.66	5.89	91.43	72.86	95.71		25	68.34	11.23	70.00	45.71	84.29
		Private	25	91.37	5.56	92.86	74.29	97.14		25	72.63	11.16	72.86	50.00	92.86

**Caption:** SD: Standard deviation; Min.: Minimum; Max: Maximum

Table 3 shows the central tendency and dispersion measures of the decoding accuracy for each school type corresponding to the school year, stimulus length and type; word/non-word. The data suggests a better decoding accuracy of the private school students in both stimuli and in all variables as well as school year. Moreover, it is fundamental to emphasize that the results show a strong effect of word length for all school years, with a more pronounced influence of such effect on non-words.

**Table 3:** Descriptive scores and comparative analysis of school types in relation to the correct answer percentage according to word length, school year and word type

**Variables**		**Words**							**Non-Words**					
**School Year**	**Length**	**School Type**	**No.**	**Average**	**SD**	**Median**	**Min.**	**Max.**	**No.**	**Average**	**SD**	**Median**	**Min.**	**Max.**
1	Mono	Public	25	14.58	17.63	7.73	0.00	46.94	25	5.87	11.18	0.00	0.00	35.29
		Private	25	39.93	25.61	39.13	0.00	112.50	25	22.38	10.84	23.44	0.00	44.44
	Di	Public	25	10.52	17.98	1.36	0.00	54.55	25	3.91	8.34	0.00	0.00	25.53
		Private	25	28.66	16.07	36.07	0.00	49.45	25	14.13	8.95	14.44	0.00	37.19
	Tri	Public	25	7.33	8.62	7.64	0.00	26.92	25	3.30	5.37	0.00	0.00	16.73
		Private	25	18.67	11.08	21.91	0.00	35.29	25	10.54	7.33	10.41	0.00	24.68
	Poli4	Public	25	3.25	7.04	0.00	0.00	25.91	25	2.08	5.31	0.00	0.00	24.19
		Private	25	8.43	8.73	6.98	0.00	29.55	25	6.83	4.23	8.22	0.00	13.02
	Poli5	Public	25	1.90	4.53	0.00	0.00	15.05	25	1.20	2.81	0.00	0.00	8.22
		Private	25	7.88	5.28	9.16	0.00	16.44	25	5.34	3.95	6.98	0.00	12.84
	Total	Public	25	7.27	8.76	3.55	0.00	23.23	25	4.19	6.26	0.00	0.00	18.46
		Private	25	14.44	8.72	14.47	0.00	29.71	25	11.33	5.32	12.40	0.00	19.66
2	Mono	Public	25	31.87	16.99	24.49	0.00	59.02	25	22.56	11.26	22.02	0.00	40.91
		Private	25	45.75	10.09	44.44	16.1	70.59	25	33.77	1.87	35.29	0.00	54.55
	Di	Public	25	22.11	11.89	19.23	0.00	48.48	25	19.61	9.74	19.13	0.00	41.03
		Private	25	30.68	9.64	32.43	0.00	44.92	25	25.09	8.00	26.17	0.00	39.25
	Tri	Public	25	17.04	10.40	16.02	0.00	38.83	25	12.83	7.20	13.08	0.00	25.79
		Private	25	24.87	8.43	24.77	0.00	47.55	25	17.74	4.87	17.98	0.00	26.02
	Poli4	Public	25	11.95	6.22	13.04	0.00	21.74	25	8.86	5.32	10.06	0.00	26.42
		Private	25	19.02	6.72	17.18	0.00	36.44	25	11.97	3.91	11.76	0.00	19.89
	Poli5	Public	25	9.01	5.34	9.16	0.00	20.25	25	7.75	4.23	8.51	0.00	13.45
		Private	25	14.03	5.34	13.74	0.00	25.61	25	8.93	3.82	9.52	0.00	13.42
	Total	Public	25	16.43	6.52	14.91	0.00	31.69	25	13.77	5.16	13.78	0.00	25.24
		Private	25	23.91	5.40	22.66	16.11	39.79	25	17.54	4.31	17.73	0.00	23.03
3	Mono	Public	25	44.42	12.17	46.39	0.00	58.06	25	35.06	5.80	34.78	25.00	51.72
		Private	25	50.97	7.63	52.94	29.03	64.29	25	38.92	8.59	38.46	16.33	56.25
	Di	Public	25	35.17	10.31	37.35	0.00	47.12	25	28.32	7.30	28.26	0.00	38.79
		Private	25	43.14	8.10	45.16	26.80	59.57	25	31.59	9.01	30.61	15.50	46.15
	Tri	Public	25	27.35	8.94	27.55	0.00	41.61	25	19.42	6.28	19.44	0.00	32.26
		Private	25	36.36	11.01	35.19	19.29	63.46	25	22.77	5.61	21.88	10.66	32.54
	Poli4	Public	25	22.43	7.50	22.16	0.00	37.84	25	13.88	5.77	13.71	0.00	24.74
		Private	25	28.50	9.89	27.99	12.07	53.04	25	19.71	6.97	17.65	9.30	40.63
	Poli5	Public	25	18.06	6.37	18.18	0.00	31.37	25	10.41	3.68	11.32	0.00	14.46
		Private	25	23.67	7.22	24.66	10.39	39.67	25	14.24	4.04	12.77	8.85	23.08
	Total	Public	25	26.59	8.05	26.50	0.00	40.45	25	18.29	5.87	18.04	0.00	28.52
		Private	25	33.48	7.99	32.65	19.33	47.02	25	23.32	5.11	22.19	13.36	34.51
4	Mono	Public	25	49.04	8.65	49.18	26.79	65.45	25	36.24	7.39	37.50	14.63	48.39
		Private	25	49.86	8.31	50.70	36.59	70.59	25	39.75	6.85	40.54	19.20	49.18
	Di	Public	25	39.68	7.88	40.19	15.31	51.85	25	30.37	7.44	30.34	9.93	46.88
		Private	25	42.37	6.20	44.44	28.77	52.33	25	34.14	8.24	33.46	13.64	48.28
	Tri	Public	25	31.51	8.26	30.16	15.23	51.01	25	23.10	5.61	24.22	10.78	33.96
		Private	25	36.53	8.42	38.04	15.99	50.00	25	28.73	7.46	28.27	16.12	44.78
	Poli4	Public	25	24.04	7.02	21.95	11.76	43.64	25	14.16	1.47	14.24	9.76	17.48
		Private	25	31.54	8.10	31.58	15.17	48.24	25	18.50	5.91	17.02	8.54	33.47
	Poli5	Public	25	23.04	7.92	21.05	7.85	38.10	25	11.82	3.01	11.97	5.94	18.83
		Private	25	27.37	6.69	27.91	12.94	36.36	25	15.68	4.68	14.94	6.82	28.57
	Total	Public	25	30.11	6.86	31.21	15.06	46.10	25	20.11	3.26	20.60	10.19	24.53
		Private	25	35.46	7.38	35.51	18.76	48.85	25	25.10	5.84	25.08	13.20	39.30
5	Mono	Public	25	43.71	6.03	43.90	34.78	54.55	25	35.35	8.61	36.14	12.50	55.56
		Private	25	48.08	9.58	48.65	24.49	76.60	25	37.17	7.92	38.10	23.08	53.73
	Di	Public	25	38.96	7.19	39.13	26.50	51.89	25	29.46	7.44	28.97	17.54	47.62
		Private	25	41.04	6.05	41.94	29.80	49.23	25	33.84	6.40	33.77	17.54	44.78
	Tri	Public	25	35.23	7.25	35.59	19.44	49.04	25	24.05	7.86	21.84	13.51	44.63
		Private	25	36.98	7.23	36.84	22.27	48.09	25	25.12	5.19	25.79	14.71	35.92
	Poli4	Public	25	28.71	7.81	29.90	15.57	43.64	25	16.57	4.59	15.58	10.99	27.66
		Private	25	30.63	8.15	29.43	17.98	44.24	25	17.94	5.17	17.65	8.53	30.95
	Poli5	Public	25	24.48	7.29	25.67	13.45	40.00	25	12.66	4.10	12.37	5.66	23.68
		Private	25	27.62	7.10	27.27	16.22	39.34	25	14.20	4.70	12.77	6.11	23.41
	Total	Public	25	32.63	6.67	33.13	21.92	44.10	25	21.31	4.55	20.49	15.25	31.86
		Private	25	34.88	6.43	35.46	24.25	45.62	25	23.47	4.53	24.54	13.74	34.71

**Caption:** SD: Standard deviation; Min.: Minimum; Max: Maximum

## DISCUSSION

The objective of the present study was to continue the validation process of the Decoding Development Monitoring Protocol (*Protocolo de Acompanhamento do Desenvolvimento da Decodificação* - PRADE) in a software format for the validity evidence stage based on the response processes, with children from public and private schools as participants.

The data evidences that the software used herein confirms the response processes’ analysis validity, since it was possible to adequately characterize the performance of children from public and private schools throughout Elementary School, regarding both each school year and the increasing positive performance of schoolchildren due to course of the schooling process. Beside characterizing each group, it also shows its differences, which were already widely acknowledged and discussed in previous studies, as well as in national and international assessments^(5,6,11)^.

On the subject of the time spent to correctly decode the stimuli, the fact that the first year of public school often presents half of the number of children than private schools indicates that there is a significant deficit in the learning of grapheme-phoneme correspondences, which can result from different scenarios. It is paramount to emphasize that BP is a medium transparency language, with a greater transparency as to the grapheme for the phoneme relationship than the other way around ^(21)^. In this perspective, some studies have shown that the delay in mastering the decoding skill in more transparent languages may be related to factors extrinsic to the child, such as the teaching methods, the family’s schooling level and literacy practices, along with social vulnerability and/or deficits in emerging literacy skills^(2,11-14,17)^.

Therefore, it is important to consider the abovementioned variables in future studies so that the software is able to characterize and monitor the students’ decoding, while simultaneously considering the individuals’ socio-environmental aspects. This becomes crucial since a meta-analysis^(24)^ aimed to verify the correlation between decoding performance at the beginning of literacy compared to at a later reading development showed evidence that decoding is a strong predictor of reading development throughout the academic life.

In other words, there is an undeniable demand for the early monitoring of children's decoding skill development through a fast, democratic instrument available in a software format for wide access, such as PRADE offers. This way, it will be possible to elaborate parent-teacher training programs and collective strategies aiming to, by analyzing the response curve to such activities, attenuate the influence of the extrinsic variables, allowing for an efficient identification of children with intrinsic difficulties and their subsequent referral for specific evaluation, as stated in the DSM-5-TR guidelines^(25)^.

With regard to the correct answer percentage and reading accuracy, the same phenomenon was observed, with a superior performance of private school children in both stimuli, for the different lengths and school years. It is worthy of mention, however, that such differences tend to display a decrease from the third school year onwards, as already pointed out in previous studies^(10,17,24,26)^, contradicting Brazil's new literacy policy, the National Commitment for Literate Children^(27)^, which recommends that Brazilian students should master basic decoding skills by the end of the second school year. Some authors argue that the greatest development in oral reading fluency measures has been observed between the first and third years of schooling, unlike what occurs between the third and fifth years, when there is a propensity for less variation in the assessment results^(10,12,17,26)^, data which was corroborated by the present study.

It should be emphasized that this development pattern finds theoretical foundation in the dual-route theory (development of automaticity of reading). Thus, as the individual learns to decode and master the written code, their performance on reading tests improves, that is, as their spelling lexicon increases, the scores related to the decoding speed tend to stabilize, with a propensity to reduce the differences among close schooling peers^(1,10,13,17)^.

The results found herein show similar development curves for all students, with improvement in all variables due to the school progression. This data confirms the cognitive science’s hypothesis of reading that states the importance of learning and consolidating decoding in the first school years to reach reading fluency and comprehension, which is the final goal of this skill acquisition^(5,6,13,14)^. It is vital to stress, however, the need for further research concerning different types of schools in different regions of Brazil for the data to be generalized, which is the next step in PRADE’s validation process. It is of fundamental importance to equally consider the performance of all students according to the stimulus length, regardless of the school type, since a strong effect of this variable was observed throughout all school years. These data are consistent with previous studies^(11,26)^ that highlighted the influence of the stimulus length on the decoding performance of Brazilian schoolchildren, once again evidencing PRADE’s effectiveness regarding the validity based on response processes.

A study postulated^(28)^ that decoding assessment measures, when used as effective markers and predictors of future reading performance, are of great value to researchers, as well as to school staff, to professionals who assess reading development and its disorders, and also to public education agencies that monitor children's performance. The researchers carried out a meta-analysis in order to investigate whether the scores considered as a cutoff for possible reading development alterations were accurate enough to perform the proper measurement of this skill in children. The results of the analysis of journals, dissertations, theses and public documents indicated a great accuracy of different studies’ cutoff scores for the identification of risk related to reading acquisition, reaffirming the decoding assessment as a valid tool for identifying potential delays in reading development^(4-6, 10-13)^.

Finally, the literature specialized in the area along with the results found herein not only corroborate and support the data found by PRADE, but also indicates that there is a demand for instruments that enable a swift, accurate and democratic evaluation of the acquisition and development of decoding throughout the early school years, which is PRADE's main purpose.

## CONCLUSION

The validity evidence stage based on the response processes of PRADE software is satisfactory in terms of characterizing different groups of the test’s target population, adequately characterizing children from public and private schools according to their school level as well as in relation to the schooling effect in the process of the decoding acquisition for all students. Therefore, this instrument is assertive and ready for the next steps of validation.
