# Supplementary material for: Software for assessment and monitoring the decoding skills development of children from the elementary school: validity based on response process
Source: Codas. 2024 Sep 13;36(5):e20230349. doi: 10.1590/2317-1782/20242023349en (PMC11404839; doi:10.1590/2317-1782/20242023349en)
Supplement: Translation

# Translation: Software para avaliação e monitoramento do desenvolvimento da decodificação em escolares do Ensino Fundamental I: validade baseada nos processos de resposta

**DOI:** 10.1590/2317-1782/20242023349pt

**Authors:** Aparecido José Couto Soares, Débora Maria Befi-Lopes

**Affiliations:** 1 Departamento de Fonoaudiologia, Escola Paulista de Medicina – EPM, Universidade Federal de São Paulo – UNIFESP – São Paulo (SP), Brasil.; 2 Departamento de Fisioterapia, Fonoaudiologia e Terapia Ocupacional, Faculdade de Medicina, Universidade de São Paulo – USP – São Paulo (SP), Brasil.

**Keywords:** Leitura, Aprendizagem, Criança, Ensino, Software

## Abstract

**Objetivo:**

Dar seguimento ao processo de validação do Protocolo de Acompanhamento do Desenvolvimento da Decodificação (PRADE) em formato de software na etapa de evidência de validade baseada nos processos de resposta.

**Método:**

Foram participantes deste estudo 250 indivíduos, sendo 125 indivíduos oriundos de escola privada e 125 indivíduos oriundos de escola pública. A avaliação foi realizada presencialmente por meio do software que hospeda as tarefas do instrumento, as quais são organizadas em decodificação de palavras e não-palavras balanceadas linguisticamente respeitando-se as regras de decodificação do Português Brasileiro. O software elabora relatório individual de desempenho de cada participante contabilizando o tempo de decodificação de cada estímulo, assim como o número de palavras decodificadas corretamente. Os dados são organizados de forma a contabilizar o tempo de decodificação correta dos estímulos, acurácia de decodificação e porcentagem de acertos. Todos os dados passaram por análise estatística por meio do software SPSS.

**Resultados:**

Os dados indicaram importante efeito da extensão de palavras e não-palavras em estudantes de escola pública e privada. Ademais, foi possível observar a evolução da decodificação, em função do ano escolar, em todas as variáveis estudadas. Em ambos os grupos observou-se forte influência das não-palavras no desempenho dos estudantes em todo o Ensino Fundamental I.

**Conclusão:**

Os dados indicam validade na análise dos processos de resposta, uma vez que foi possível caracterizar adequadamente o desempenho de crianças de escola pública e privada em todo o Ensino Fundamental I, caracterizando cada grupo, bem como suas diferenças conforme o avanço da escolaridade.

## INTRODUÇÃO

A avaliação da decodificação por meio da leitura em voz alta é a medida mais utilizada para monitorar a aquisição e o progresso das crianças, tanto no que diz respeito às avaliações escolares, quanto para a verificação de efetividade de programas de estimulação e/ou intervenção^(1,2)^. Além disso, o resultado da avaliação da decodificação oral é de tamanha importância que é considerado um preditor fundamental do desempenho do indivíduo em leitura. Nos Estados Unidos, por exemplo, as medidas de avaliação da leitura oral são analisadas pelo *Federal Education Department* para acompanhar o desenvolvimento acadêmico das crianças e para o desenvolvimento de programas de estimulação precoce^(1,2)^.

A leitura oral de palavras isoladas é, sem dúvida, a tarefa mais utilizada para avaliar a proficiência do indivíduo em decodificação. Um dos motivos é o fato de que esta tarefa isola as pistas de contexto ou visuais (pictóricas) e, dessa maneira, avalia estritamente a decodificação^(2)^. Os modelos de avaliação de leitura são fortemente baseados na teoria de dupla rota, uma vez que utilizam palavras e não palavras^(2)^. É importante ressaltar que esforços têm sido realizados para criar e validar testes de leitura para falantes do Português Brasileiro (PB)^(3-6)^, porém, grande parte deles estão mais voltados para a leitura e seus processos e não para a decodificação isolada, além utilizarem pouco a investigação das propriedades psicométricas dos testes^(7-9)^.

Segundo a literatura^(10)^, crianças que demoram ou falham em adquirir a habilidade de reconhecimento automático de palavras podem estar em risco para diferentes alterações de aprendizagem do código escrito. Entretanto, apesar dos avanços em relação ao estudo da leitura quanto aos seus parâmetros e habilidades subjacentes, no Brasil, ainda é reduzido o número de procedimentos validados para a devida avaliação e conduta dos possíveis transtornos de leitura e de aprendizagem^(3-9)^. Não obstante, grande parte dos instrumentos não está em forma de *software* ou aplicativo, o que pode não apenas modernizar o processo de avaliação da decodificação, mas também prover dados mais fidedignos em relação ao tempo despendido na decodificação, número/porcentagem de acertos ou acurácia, quantidade de palavras lidas corretamente por minuto ou em um minuto, medidas consideradas padrão-ouro em provas de decodificação no exterior. Como exceção, há o aplicativo EduEdu® que possui atividades de avaliação e monitoramento da aprendizagem do código escrito como um todo, entretanto, sem a adequada validação científica^(11)^.

Em um país com a realidade educacional como a do Brasil, na qual é comum os escolares apresentarem alguma dificuldade para ler ou escrever, torna-se imprescindível a caracterização da condição leitora das crianças precocemente, principalmente na fase da aprendizagem da decodificação. Isso porque de acordo com a última avaliação do Programme *for International Student Assessment* – PISA, o Brasil permaneceu com altos índices de fracasso escolar^(12)^. Segundo os dados publicados, o país permanece há mais de dez anos entre os países com pior desempenho. Ainda de acordo com este último relatório, as dificuldades em leitura enfrentadas pelos escolares brasileiros têm início na educação básica, ou seja, no domínio da decodificação, e interferem na consolidação da alfabetização. Estas dificuldades, quando mal identificadas, se tornam crônicas, levando o estudante a baixa *performance* ao longo de toda a sua trajetória escolar^(12)^.

Conforme já mencionado, a ausência de procedimentos adequadamente validados impede que os profissionais envolvidos diretamente no trabalho com escolares (fonoaudiólogos, professores e psicólogos) tenham parâmetros adequados para a identificação das dificuldades e das alterações na decodificação e, consequentemente, possam desenvolver estratégias de estimulação mais adequadas, inclusive em sala de aula^(10)^. Em países desenvolvidos esta realidade é bastante diferente, havendo o uso de testes validados para avaliação da decodificação, fluência e compreensão da leitura. Geralmente, estes testes são compostos por listas de palavras e textos que devem ser lidos pelas crianças em um intervalo pré-determinado de tempo^(10,13,14)^.

Sendo assim, o desenvolvimento de um instrumento brasileiro de avaliação da decodificação, que possa ser utilizado como ferramenta para monitorar o desenvolvimento desta habilidade, se mostra fundamental tanto em âmbito clínico como educacional. Ademais, sendo tal instrumento em formato de *software,* no qual se pode aferir o desempenho com mais fidedignidade, que esteja adequadamente validado com suas características psicométricas analisadas e com acesso livre para fonoaudiólogos, psicopedagogos, pais, professores e demais profissionais que trabalham na aprendizagem, é de extrema necessidade. Assim, pode se construir uma ampla rede de apoio e de vigilância do desenvolvimento da decodificação que tem como característica a abordagem multidisciplinar e a construção de relações mais sólidas entre os profissionais e a família da criança^(15)^. Importante ressaltar que instrumentos de vigilância devem ser aplicados em indivíduos saudáveis e não-saudáveis, visando identificar aqueles que apresentam sinais de risco quanto ao desenvolvimento de determinada condição ou doença, para que a identificação precoce traga melhores resultados^(16)^. Para que seja efetivo, o instrumento deve ser validado, de simples compreensão e aplicação, possuir reprodutibilidade e acurácia^(16)^.

Considerando e exposto, o objetivo deste estudo é dar seguimento ao processo de validação do Protocolo de Acompanhamento do Desenvolvimento da Decodificação (PRADE), em formato de *software,* na etapa de evidência de validade baseada nos processos de resposta, uma vez que as etapas anteriores encontram-se publicadas e referenciadas^(17-19)^.

## MÉTODO

Essa pesquisa foi aprovada pelo Comitê de Ética em Pesquisa da Faculdade de Medicina da Universidade de São Paulo (FMUSP) (CEP nº 2.262.300). Estudo prospectivo que seguiu os princípios do *Standards for Educational and Psychological Testing* (SEPT)^(20)^, diretriz proposta por organizações americanas que compila recomendações e definições fundamentais quanto aos aspectos psicométricos envolvidos desde a elaboração até a interpretação dos testes, além das diferentes etapas necessárias para validação de um procedimento. Os procedimentos de coleta de dados tiveram início somente após a assinatura do Termo de Consentimento Livre e Esclarecido pela escola envolvida no estudo e pelos pais/responsáveis, além da assinatura do Termo de Assentimento pelas crianças.

### Casuística

Foram participantes deste estudo 250 indivíduos, sendo 125 indivíduos oriundos de escola privada e 125 indivíduos oriundos de escola pública. Os dois grupos, por sua vez, foram subdivididos em cinco grupos de tamanho igual (n = 25), de acordo com o ano escolar, ou seja, do primeiro ao quinto ano escolar. Para serem incluídas no estudo, as crianças deveriam atender aos seguintes critérios: estar regularmente matriculada no Ensino Fundamental I; ausência de queixas/ indicadores de alterações auditivas ou visuais; ausência de indicativos de transtornos neurológicos ou cognitivos; ausência de retenção no histórico escolar; ausência de alteração fonológica e de linguagem oral, verificados por meio de triagem fonoaudiológica.

A fim de garantir que a amostra do estudo fosse composta por crianças com diferentes perfis acadêmicos e para evitar que fossem indicados para participação um perfil único de crianças, fosse aquelas com melhor desempenho acadêmico ou aquelas com maior dificuldade em aprender a decodificar, optou-se por utilizar a amostragem aleatória estratificada para a seleção dos participantes. Dessa forma, as crianças foram numeradas, inicialmente, de 1 a 150, em ordem crescente de acordo com o ano escolar, em ambas as escolas. Em seguida, estes números foram utilizados para selecionar, aleatoriamente, a amostra final do estudo, caracterizada a seguir.

Dessa forma, o grupo de crianças da escola pública foi composto da seguinte maneira: 25 crianças do 1º ano (12 meninas e 13 meninos; média de idade de 6,56; 25 crianças do 2º ano escolar (14 meninas e 11 meninos; média de idade de 7,47); 25 crianças do 3º ano (11 meninas e 14 meninos; média de idade de 8,65); 25 crianças do 4º ano (12 meninas e 13 meninos; média 38 de idade de 9,64); 25 crianças do 5º ano (10 meninas e 15 meninos; média de idade de 10,52 s). O grupo de crianças da escola privada constituiu-se da seguinte forma: 25 crianças do 1º ano (14 meninos e 11 meninas; média de idade de 6,60); 25 crianças do 2º ano (12 meninas e 14 meninos; média de idade de 7,25; 25 crianças do 3º ano (11 meninas e 14 meninos; média de idade de 8,52 anos); 25 crianças do 4º ano (14 meninas e 11 meninos; média de idade de 9,56 anos); 25 crianças do 5º ano (11 meninas e 14 meninos; média de idade de 10,52 anos).

#### Procedimentos

Considerando que este estudo faz parte do processo de validação do PRADE, é importante salientar que as etapas de evidência de validade baseada no conteúdo do teste; delimitação da população-alvo; elaboração dos itens; análise de juízes com expertise na área; determinação do tamanho amostral; procedimentos para verificar se a população entende os itens do teste; aplicação do teste em uma amostra da população-alvo; análise dos dados e correlações foram realizadas em estudo anterior e publicado em periódico internacional^(17)^ confirmando a validade destes aspectos.

Sendo assim, segue-se para a etapa de evidência de validade baseada nos processos de resposta, que se caracteriza por analisar o desempenho de diferentes estratos da população-alvo na aplicação da tarefa, buscando-se entender os processos envolvidos no padrão de respostas^(21)^. Importante salientar que fases desta etapa já foram realizadas em estudos anteriores com crianças com transtorno do desenvolvimento da linguagem (TDL), analisando-se o desempenho desta população no teste aqui apresentado em dois estudos publicados em periódicos indexados no Web of Science com fator de impacto relevante^(18,19)^. Os dados evidenciaram diferença no desempenho dos sujeitos com TDL quando comparado aos seus pares típicos, indicando importante validade discriminante do instrumento entre crianças típicas e aquelas com TDL. Entretanto, há a necessidade de se confirmar este último aspecto em estudos futuros e com diferentes populações.

Cabe ressaltar ainda que, quando se trata de elaboração de materiais para verificar aspectos da linguagem escrita no Brasil, deve-se levar em consideração o histórico de desempenho sofrível em avaliações nacionais e internacionais, índices de alfabetização e analfabetismo funcional alarmantes, principalmente em estudantes oriundos de escolas públicas e aqueles na interseccionalidade escola pública somada à vulnerabilidade social. Neste sentido, faz-se necessária a verificação da efetividade do teste em caracterizar adequadamente o desempenho de estudantes de escolas públicas e privadas.

O PRADE consiste de palavras balanceadas linguisticamente conforme as regras de decodificação do Português PB^(22)^ respeitando-se também a variação de extensão de palavras de mono a polissílabos para crianças desta faixa escolar^(23)^. A prova também conta com não-palavras que foram derivadas das palavras reais e que também seguem as regras de decodificação do PB, bem como a variação de mono a polissílabos^(23)^.

A avaliação foi realizada presencialmente por meio do *software* que hospeda as tarefas do PRADE, o Psychopy®, no contra-turno escolar dos participantes. Ressalta-se que a coleta de dados ocorreu no segundo semestre do ano letivo dos participantes, pois assim garante-se que o perfil de habilidades acadêmicas esteja condizente com o ano escolar dos indivíduos, principalmente no que se refere a estudantes do primeiro ano escolar. Ao iniciar o *software* aparece uma tela inicial dando boas vindas ao participante e explicando a forma como o procedimento será realizado (Figura 1).

**Figura 1:** Tela inicial do PRADE contendo as boas vindas e instruções

O procedimento inicia-se pelas palavras reais que são apresentadas randomicamente em letra formato arial, caixa alta, nº 20 escritas em branco em uma tela com fundo cinza (Figura 2).

**Figura 2:** Exemplo de como os estímulos aparecem no PRADE

Durante a decodificação o avaliador aperta o número 0 para cada decodificação errada e o número 1 para cada decodificação correta. Cabe ressaltar que foi considerado decodificação correta aquela em que foram respeitadas as relações grafofonêmicas, as regras ortográficas e adequada tonicidade em estímulos com sinais diacríticos^(22)^. Em seguida, inicia-se a decodificação das não-palavras ao mostrar ao participante uma nova tela contendo as novas instruções (Figura 3).

**Figura 3:** Tela de início da decodificação de não-palavras

O procedimento de avaliação da decodificação das não-palavras ocorre igualmente ao descrito na decodificação de palavras. Ressalta-se que para a caracterização da decodificação correta ou incorreta para as pseudopalavras considerou-se estritamente a capacidade do participante em usar as regras de decodificação do PB, inclusive para aquelas que continham algum sinal diacrítico^(22)^. Cabe salientar que para assegurar que a tarefa fosse plenamente entendida, o avaliador leu para os participantes as instruções contidas na tela. Ademais, cada criança levou em média 2 minutos e 30 segundos para a realização da tarefa inteira.

O *software* utilizado elabora relatório individual de desempenho de cada participante contabilizando o tempo de decodificação de cada estímulo, desde os monossílabos até os polissílabos, assim como o número de palavras decodificadas corretamente. Em seguida os dados foram tabulados considerando o tempo de decodificação de palavras corretas, a porcentagem de acertos por extensão do estímulo (de mono a polissílabo) e seus valores totais, bem como a acurácia de decodificação, ou seja, a quantidade de palavras/não-palavras decodificadas corretamente por minuto. Foram também considerados a extensão do estímulo e seus valores totais, tanto para palavras quanto para não-palavras. É fundamental destacar que na análise do tempo despendido para a decodificação correta de palavras e não palavras, os participantes que não decodificaram corretamente nenhum estímulo, foram excluídos. Todos os dados passaram por análise estatística por meio do *software* SPSS Statistics, versão 28.0 (IBM Corp., Armonk, NY, EUA).

## RESULTADOS

A Tabela 1 apresenta as medidas de tendência central e de dispersão do tempo de decodificação para cada tipo de escola de acordo com o ano escolar, extensão da palavra e tipo de estímulo, palavra/não-palavra. Os dados indicam maior número de crianças do primeiro ano de escola privada que conseguiram efetivamente decodificar palavras e não-palavras, diminuindo-se o número de participantes de escola pública em função do tamanho do estímulo. A influência da extensão do estímulo é evidente nos demais anos escolares, assim como uma aproximação maior do tempo de decodificação de participantes de escola pública e privada, bem como o aumento do número de crianças que conseguiram realizar a tarefa a partir do segundo ano. Com o decorrer do processo de escolarização chega-se ao equilíbrio do número de participantes e proximidade nos tempos de decodificação correta dos estímulos a partir do terceiro ano escolar, estabilizando-se em todos os participantes a partir de então.

**Tabela 1:** Valores descritivos e análise comparativa dos tipos de escola em relação ao tempo de leitura de palavras corretas de acordo com a extensão da palavra, ano escolar e tipo de palavras

**Variáveis**		**Palavras**								**Não-Palavras**					
**Ano escolar**	**Extensão**	**Escola**	**n**	**Média**	**DP**	**Mediana**	**Mín.**	**Máx.**		**n**	**Média**	**DP**	**Mediana**	**Mín.**	**Máx.**
1	Mono	Pública	14	6,16	3,01	7,04	1,40	12,01		6	9,65	2,87	8,65	6,50	14,60
		Privada	23	9,28	8,49	7,00	,90	45,80		22	10,39	3,95	9,95	2,10	19,50
	Di	Pública	14	28,34	16,01	32,75	1,10	51,30		5	27,64	3,34	28,20	22,20	31,20
		Privada	20	20,12	7,60	20,20	3,10	36,20		20	40,88	11,36	39,80	21,20	59,10
	Tri	Pública	13	17,20	14,91	12,10	5,50	55,52		8	38,84	23,75	49,20	7,60	68,20
		Privada	20	31,04	9,81	34,20	10,10	45,30		20	64,98	27,32	63,95	33,40	142,90
	Poli4	Pública	5	21,60	10,05	25,30	5,20	30,10		5	30,28	17,88	22,20	12,40	55,30
		Privada	19	72,51	32,62	67,10	23,40	137,40		19	50,79	24,66	52,30	8,20	98,20
	Poli5	Pública	4	26,63	8,32	29,90	14,50	32,20		4	26,08	14,25	26,70	8,20	42,70
		Privada	19	30,86	14,43	32,10	8,20	66,00		17	28,18	13,25	25,70	8,30	49,60
	Total	Pública	15	61,41	47,66	49,70	4,60	161,91		9	84,72	75,60	103,90	6,50	192,80
		Privada	23	145,43	60,79	157,85	6,10	262,40		22	172,27	74,47	171,00	3,20	289,80
2	Mono	Pública	23	9,24	3,55	9,20	1,20	16,50		23	10,10	2,87	10,10	4,50	17,20
		Privada	25	7,96	1,87	7,50	5,10	14,90		24	8,66	1,64	8,50	4,20	11,60
	Di	Pública	24	28,03	10,76	28,90	7,50	57,90		22	31,19	9,75	32,85	4,10	54,50
		Privada	24	24,65	5,44	24,65	15,40	38,20		24	30,12	7,81	29,60	19,80	48,20
	Tri	Pública	21	40,99	11,41	41,20	22,20	68,90		21	45,25	13,51	44,60	19,20	86,70
		Privada	24	36,89	10,52	34,70	19,20	59,20		24	43,35	9,12	41,75	22,30	59,20
	Poli4	Pública	21	38,70	9,76	39,80	14,90	64,50		21	39,43	13,76	38,90	12,20	61,20
		Privada	24	35,88	8,84	35,70	21,70	55,30		24	33,66	11,86	36,35	5,10	53,20
	Poli5	Pública	21	31,72	8,65	32,30	9,20	48,20		20	21,67	7,71	21,20	6,70	35,70
		Privada	24	25,03	9,30	24,05	5,70	54,20		22	21,95	11,72	22,40	6,30	43,80
	Total	Pública	24	134,37	52,42	147,25	8,20	208,80		23	136,08	51,19	149,10	8,60	221,80
		Privada	25	125,51	35,25	131,00	14,90	177,70		24	135,91	32,63	140,40	70,80	192,90
3	Mono	Pública	24	7,66	1,41	7,55	4,70	11,30		25	8,03	2,09	8,80	2,40	11,70
		Privada	25	7,11	1,45	6,70	5,50	12,40		25	7,89	2,20	7,80	3,90	14,70
	Di	Pública	24	24,56	5,78	23,10	18,70	44,90		24	28,65	4,81	28,05	19,20	39,10
		Privada	25	20,58	4,09	19,20	14,10	32,10		25	28,56	8,78	26,50	19,50	54,20
	Tri	Pública	24	40,81	7,14	39,50	27,40	56,70		24	47,39	7,88	45,95	34,20	61,20
		Privada	25	36,25	10,84	34,10	20,80	62,20		25	40,26	9,63	37,20	28,20	57,20
	Poli4	Pública	24	37,18	8,58	37,20	22,20	57,20		24	63,16	104,56	40,85	22,10	550,70
		Privada	25	33,03	10,07	29,80	18,10	55,40		25	28,26	8,30	29,10	12,30	49,50
	Poli5	Pública	24	22,66	4,30	22,70	15,30	32,20		23	25,53	10,86	24,20	6,20	55,20
		Privada	25	19,33	5,52	18,60	12,10	31,20		25	20,47	8,14	19,60	6,10	33,90
	Total	Pública	24	132,87	23,21	133,10	97,90	200,20		24	171,93	103,19	155,30	92,50	639,70
		Privada	25	116,31	25,06	115,50	85,50	171,20		25	125,44	26,31	127,10	78,10	176,40
4	Mono	Pública	25	7,06	1,38	7,10	5,10	11,20		25	7,38	1,83	6,90	4,50	11,70
		Privada	25	7,12	1,06	7,00	5,10	8,90		25	7,94	1,83	7,60	5,00	13,20
	Di	Pública	25	22,29	4,63	21,20	16,20	39,20		25	23,48	4,96	22,40	14,50	37,50
		Privada	25	21,13	2,87	20,70	17,20	29,20		25	26,76	6,70	26,40	17,40	52,80
	Tri	Pública	25	38,56	8,14	38,10	24,70	52,90		25	36,08	8,99	33,40	22,30	60,70
		Privada	25	37,06	9,96	34,70	26,40	66,20		25	35,77	8,64	34,50	23,20	53,20
	Poli4	Pública	25	34,51	7,91	32,60	22,00	50,10		25	37,86	8,43	41,20	12,30	48,20
		Privada	25	31,50	9,16	30,00	19,90	63,30		25	35,86	10,50	32,20	22,40	59,10
	Poli5	Pública	25	20,27	6,45	20,70	12,30	38,20		25	24,52	9,18	24,50	10,10	41,20
		Privada	25	18,54	5,80	16,50	13,20	37,10		25	22,86	6,78	22,20	14,50	45,20
	Total	Pública	25	122,68	21,03	121,30	83,30	167,30		25	129,30	23,20	130,90	84,90	187,40
		Privada	25	115,35	27,26	105,90	82,30	204,70		25	129,19	24,83	124,50	93,60	181,80
5	Mono	Pública	25	7,74	1,18	7,70	5,70	10,30		25	8,60	3,11	8,30	4,10	19,20
		Privada	25	7,56	1,95	7,30	4,70	14,70		25	8,59	2,54	7,80	4,70	15,60
	Di	Pública	25	23,11	4,23	23,00	17,90	34,10		25	25,98	6,03	25,90	15,20	40,20
		Privada	25	22,28	3,63	21,10	17,10	30,20		25	25,82	4,80	25,00	18,00	38,10
	Tri	Pública	25	36,08	7,47	35,40	20,80	53,20		25	40,31	10,42	41,20	22,20	55,20
		Privada	25	34,88	7,98	33,20	21,20	54,60		25	39,32	9,35	38,20	23,20	61,20
	Poli4	Pública	25	32,69	7,94	30,50	22,00	50,10		25	38,90	10,07	39,10	23,40	63,20
		Privada	25	31,24	8,11	29,10	21,70	53,40		25	35,84	12,93	34,20	12,20	79,20
	Poli5	Pública	25	19,77	4,97	18,70	12,00	28,60		25	23,91	7,51	22,30	8,10	46,10
		Privada	25	17,37	4,72	16,40	10,70	29,30		25	23,78	8,66	21,10	6,20	49,10
	Total	Pública	25	119,39	21,50	114,10	88,50	168,80		25	137,70	24,49	133,60	99,80	193,40
		Privada	25	113,34	20,49	108,40	86,80	160,80		25	133,35	27,59	132,40	90,00	209,40

**Legenda:** DP: Desvio padrão; Mín.: Mínimo; Máx.: Máximo

A Tabela 2 apresenta as medidas de tendência central e de dispersão da porcentagem de acertos para cada tipo de escola de acordo com o ano escolar, extensão da palavra e tipo de estímulo, palavra/não-palavra. Mais uma vez, os dados evidenciam a influência da extensão do estímulo na porcentagem de acertos em crianças de ambas as escolas, em todos os anos escolares, principalmente em relação às não-palavras. Importante ressaltar que os estudantes de escola privada apresentam maior porcentagem de acertos em todos os anos escolares e em todas as extensões. Os dados também evidenciam a semelhança de desempenho dos estudantes a partir do terceiro ano escolar, indicando estabilização de desempenho nos anos escolares posteriores.

**Tabela 2:** Valores descritivos e análise comparativa dos tipos de escola em relação à porcentagem de acertos de acordo com a extensão da palavra, ano escolar e tipo de palavra

**Variáveis**				**Palavras**						**Não-Palavras**					
**Ano escolar**	**Extensão**	**Escola**	**n**	**Média**	**DP**	**Mediana**	**Mín.**	**Máx.**		**n**	**Média**	**DP**	**Mediana**	**Mín.**	**Máx.**
1	Mono	Pública	25	23,33	34,36	16,67	0,00	100		25	15,33	29,23	0,00	0,00	83,33
		Privada	25	74,00	35,38	83,33	0,00	100		25	62,00	32,46	66,67	0,00	100
	Di	Pública	25	15,75	26,34	6,25	0,00	87,50		25	11,50	24,98	0,00	0,00	75,00
		Privada	25	57,00	34,63	68,75	0,00	93,75		25	58,25	35,07	75,00	0,00	93,75
	Tri	Pública	25	12,18	22,87	4,55	0,00	86,36		25	10,91	21,80	0,00	0,00	68,18
		Privada	25	43,82	29,45	50,00	0,00	86,36		25	45,64	28,85	54,55	0,00	86,36
	Poli4	Pública	25	8,00	19,97	,00	0,00	81,25		25	5,50	12,67	0,00	0,00	43,75
		Privada	25	46,00	33,07	56,25	0,00	93,75		25	36,75	29,50	37,50	0,00	87,50
	Poli5	Pública	25	10,50	26,19	,00	0,00	100		25	6,50	16,97	0,00	0,00	62,50
		Privada	25	48,00	34,74	50,00	0,00	100		25	32,50	31,87	37,50	0,00	100
	Total	Pública	25	12,46	23,20	4,29	0,00	85,71		25	9,37	19,49	0,00	0,00	61,43
		Privada	25	49,09	30,62	62,86	0,00	90,00		25	45,09	28,23	57,14	0,00	81,43
2	Mono	Pública	25	72,00	30,32	83,33	0,00	100		25	60,00	27,22	66,67	0,00	100
		Privada	25	96,67	9,62	100	66,67	100		25	79,33	22,71	83,33	0,00	100
	Di	Pública	25	62,50	28,30	62,50	0,00	100		25	62,75	32,05	68,75	0,00	100
		Privada	25	76,50	21,29	87,50	0,00	93,75		25	76,75	24,44	81,25	0,00	100
	Tri	Pública	25	51,09	29,32	54,55	0,00	95,45		25	43,27	25,62	45,45	0,00	86,36
		Privada	25	68,36	24,81	77,27	0,00	100		25	58,00	19,21	63,64	0,00	81,82
	Poli4	Pública	25	49,25	29,50	50,00	0,00	93,75		25	35,75	23,21	37,50	0,00	87,50
		Privada	25	69,75	24,18	75,00	0,00	100		25	42,75	21,70	43,75	0,00	75,00
	Poli5	Pública	25	56,50	31,69	62,50	0,00	100		25	36,00	26,59	37,50	0,00	100
		Privada	25	70,50	27,21	75,00	0,00	100		25	40,50	28,48	37,50	0,00	87,50
	Total	Pública	25	54,23	27,42	57,14	0,00	91,43		25	45,37	24,85	48,57	0,00	88,57
		Privada	25	71,26	20,60	74,29	5,71	92,86		25	56,97	19,74	60,00	0,00	81,43
3	Mono	Pública	25	92,00	21,04	100	0,00	100		25	77,33	18,56	83,33	16,67	100
		Privada	25	98,00	5,53	100	83,33	100		25	82,00	17,29	83,33	50,00	100
	Di	Pública	25	86,50	20,06	87,50	0,00	100		25	83,25	20,23	87,50	0,00	100
		Privada	25	89,50	6,68	87,50	81,25	100		25	87,50	11,69	87,50	50,00	100
	Tri	Pública	25	81,45	19,28	81,82	0,00	100		25	68,36	19,48	68,18	0,00	90,91
		Privada	25	92,18	8,85	95,45	54,55	100		25	66,73	11,86	68,18	31,82	86,36
	Poli4	Pública	25	82,75	20,19	87,50	0,00	100		25	59,50	16,74	62,50	0,00	81,25
		Privada	25	89,00	9,76	87,50	62,50	100		25	56,75	18,92	62,50	12,50	87,50
	Poli5	Pública	25	81,50	20,45	87,50	0,00	100		25	55,00	28,18	62,50	0,00	100
		Privada	25	89,00	15,44	100	50,00	100		25	60,50	24,12	62,50	12,50	87,50
	Total	Pública	25	81,49	18,55	85,71	0,00	95,71		25	66,97	17,43	70,00	0,00	85,71
		Privada	25	88,34	6,26	90,00	65,71	95,71		25	67,89	12,17	70,00	37,14	82,86
4	Mono	Pública	25	93,33	8,33	100	83,33	100		25	72,67	17,27	83,33	33,33	100
		Privada	25	96,67	8,33	100	66,67	100		25	85,33	13,88	83,33	66,67	100
	Di	Pública	25	88,75	8,84	93,75	62,50	100		25	72,75	18,39	75,00	31,25	100
		Privada	25	91,75	7,59	93,75	75,00	100		25	90,75	11,71	93,75	50,00	100
	Tri	Pública	25	87,82	11,49	90,91	59,09	100		25	61,27	13,89	63,64	27,27	81,82
		Privada	25	96,73	5,33	100	77,27	100		25	74,36	12,71	72,73	45,45	100
	Poli4	Pública	25	83,00	16,19	87,50	37,50	100		25	56,50	14,93	62,50	12,50	81,25
		Privada	25	96,75	4,46	100	87,50	100		25	65,25	15,84	68,75	43,75	100
	Poli5	Pública	25	88,50	12,97	87,50	50,00	100		25	60,00	23,11	62,50	12,50	87,50
		Privada	25	98,50	4,15	100	87,50	100		25	71,00	16,82	75,00	37,50	100
	Total	Pública	25	84,97	9,04	87,14	60,00	95,71		25	61,89	13,48	65,71	22,86	77,14
		Privada	25	93,03	3,72	94,29	81,43	97,14		25	74,46	9,65	75,71	52,86	91,43
5	Mono	Pública	25	92,67	9,72	100	66,67	100		25	78,67	15,61	83,33	50,00	100
		Privada	25	96,67	6,80	100	83,33	100		25	84,67	14,37	83,33	50,00	100
	Di	Pública	25	91,00	6,01	93,75	75,00	100		25	76,75	14,38	75,00	43,75	100
		Privada	25	93,25	5,68	93,75	81,25	100		25	88,75	11,69	93,75	56,25	100
	Tri	Pública	25	92,73	9,00	95,45	63,64	100		25	69,64	14,05	72,73	22,73	95,45
		Privada	25	94,18	10,21	100	54,55	100		25	72,55	13,67	72,73	45,45	100
	Poli4	Pública	25	92,00	8,75	93,75	62,50	100		25	65,50	17,60	68,75	31,25	87,50
		Privada	25	93,75	7,22	93,75	75,00	100		25	63,50	17,09	68,75	18,75	87,50
	Poli5	Pública	25	94,00	9,63	100	62,50	100		25	63,00	24,60	62,50	12,50	100
		Privada	25	94,00	8,93	100	75,00	100		25	68,00	24,49	75,00	12,50	100
	Total	Pública	25	89,66	5,89	91,43	72,86	95,71		25	68,34	11,23	70,00	45,71	84,29
		Privada	25	91,37	5,56	92,86	74,29	97,14		25	72,63	11,16	72,86	50,00	92,86

**Legenda:** DP: Desvio padrão; Mín.: Mínimo; Máx.: Máximo

A Tabela 3 apresenta as medidas de tendência central e de dispersão da acurácia de decodificação para cada tipo de escola de acordo com o ano escolar, extensão e tipo de estímulo, palavra/pseudopalavra. Os dados evidenciam melhor acurácia de decodificação em estudantes de escola privada em ambos os estímulos e em todas as variáveis e ano escolar. Ademais, é de fundamental importância ressaltar que os resultados evidenciam forte efeito da extensão da palavra em todos os anos escolares, com influência mais pronunciada de tal efeito nas não-palavras.

**Tabela 3:** Valores descritivos e análise comparativa dos tipos de escola em relação à acurácia de leitura de acordo com a extensão da palavra, ano escolar e tipo de palavra

**Variáveis**		**Palavras**							**Não-Palavras**					
**Ano escolar**	**Extensão**	**Escola**	**n**	**Média**	**DP**	**Mediana**	**Mín.**	**Máx.**	**n**	**Média**	**DP**	**Mediana**	**Mín.**	**Máx.**
1	Mono	Pública	25	14,58	17,63	7,73	0,00	46,94	25	5,87	11,18	0,00	0,00	35,29
		Privada	25	39,93	25,61	39,13	0,00	112,50	25	22,38	10,84	23,44	0,00	44,44
	Di	Pública	25	10,52	17,98	1,36	0,00	54,55	25	3,91	8,34	0,00	0,00	25,53
		Privada	25	28,66	16,07	36,07	0,00	49,45	25	14,13	8,95	14,44	0,00	37,19
	Tri	Pública	25	7,33	8,62	7,64	0,00	26,92	25	3,30	5,37	0,00	0,00	16,73
		Privada	25	18,67	11,08	21,91	0,00	35,29	25	10,54	7,33	10,41	0,00	24,68
	Poli4	Pública	25	3,25	7,04	0,00	0,00	25,91	25	2,08	5,31	0,00	0,00	24,19
		Privada	25	8,43	8,73	6,98	0,00	29,55	25	6,83	4,23	8,22	0,00	13,02
	Poli5	Pública	25	1,90	4,53	0,00	0,00	15,05	25	1,20	2,81	0,00	0,00	8,22
		Privada	25	7,88	5,28	9,16	0,00	16,44	25	5,34	3,95	6,98	0,00	12,84
	Total	Pública	25	7,27	8,76	3,55	0,00	23,23	25	4,19	6,26	0,00	0,00	18,46
		Privada	25	14,44	8,72	14,47	0,00	29,71	25	11,33	5,32	12,40	0,00	19,66
2	Mono	Pública	25	31,87	16,99	24,49	0,00	59,02	25	22,56	11,26	22,02	0,00	40,91
		Privada	25	45,75	10,09	44,44	16,1	70,59	25	33,77	10,87	35,29	0,00	54,55
	Di	Pública	25	22,11	11,89	19,23	0,00	48,48	25	19,61	9,74	19,13	0,00	41,03
		Privada	25	30,68	9,64	32,43	0,00	44,92	25	25,09	8,00	26,17	0,00	39,25
	Tri	Pública	25	17,04	10,40	16,02	0,00	38,83	25	12,83	7,20	13,08	0,00	25,79
		Privada	25	24,87	8,43	24,77	0,00	47,55	25	17,74	4,87	17,98	0,00	26,02
	Poli4	Pública	25	11,95	6,22	13,04	0,00	21,74	25	8,86	5,32	10,06	0,00	26,42
		Privada	25	19,02	6,72	17,18	0,00	36,44	25	11,97	3,91	11,76	0,00	19,89
	Poli5	Pública	25	9,01	5,34	9,16	0,00	20,25	25	7,75	4,23	8,51	0,00	13,45
		Privada	25	14,03	5,34	13,74	0,00	25,61	25	8,93	3,82	9,52	0,00	13,42
	Total	Pública	25	16,43	6,52	14,91	0,00	31,69	25	13,77	5,16	13,78	0,00	25,24
		Privada	25	23,91	5,40	22,66	16,11	39,79	25	17,54	4,31	17,73	0,00	23,03
3	Mono	Pública	25	44,42	12,17	46,39	0,00	58,06	25	35,06	5,80	34,78	25,00	51,72
		Privada	25	50,97	7,63	52,94	29,03	64,29	25	38,92	8,59	38,46	16,33	56,25
	Di	Pública	25	35,17	10,31	37,35	0,00	47,12	25	28,32	7,30	28,26	0,00	38,79
		Privada	25	43,14	8,10	45,16	26,80	59,57	25	31,59	9,01	30,61	15,50	46,15
	Tri	Pública	25	27,35	8,94	27,55	0,00	41,61	25	19,42	6,28	19,44	0,00	32,26
		Privada	25	36,36	11,01	35,19	19,29	63,46	25	22,77	5,61	21,88	10,66	32,54
	Poli4	Pública	25	22,43	7,50	22,16	0,00	37,84	25	13,88	5,77	13,71	0,00	24,74
		Privada	25	28,50	9,89	27,99	12,07	53,04	25	19,71	6,97	17,65	9,30	40,63
	Poli5	Pública	25	18,06	6,37	18,18	0,00	31,37	25	10,41	3,68	11,32	0,00	14,46
		Privada	25	23,67	7,22	24,66	10,39	39,67	25	14,24	4,04	12,77	8,85	23,08
	Total	Pública	25	26,59	8,05	26,50	0,00	40,45	25	18,29	5,87	18,04	0,00	28,52
		Privada	25	33,48	7,99	32,65	19,33	47,02	25	23,32	5,11	22,19	13,36	34,51
4	Mono	Pública	25	49,04	8,65	49,18	26,79	65,45	25	36,24	7,39	37,50	14,63	48,39
		Privada	25	49,86	8,31	50,70	36,59	70,59	25	39,75	6,85	40,54	19,20	49,18
	Di	Pública	25	39,68	7,88	40,19	15,31	51,85	25	30,37	7,44	30,34	9,93	46,88
		Privada	25	42,37	6,20	44,44	28,77	52,33	25	34,14	8,24	33,46	13,64	48,28
	Tri	Pública	25	31,51	8,26	30,16	15,23	51,01	25	23,10	5,61	24,22	10,78	33,96
		Privada	25	36,53	8,42	38,04	15,99	50,00	25	28,73	7,46	28,27	16,12	44,78
	Poli4	Pública	25	24,04	7,02	21,95	11,76	43,64	25	14,16	1,47	14,24	9,76	17,48
		Privada	25	31,54	8,10	31,58	15,17	48,24	25	18,50	5,91	17,02	8,54	33,47
	Poli5	Pública	25	23,04	7,92	21,05	7,85	38,10	25	11,82	3,01	11,97	5,94	18,83
		Privada	25	27,37	6,69	27,91	12,94	36,36	25	15,68	4,68	14,94	6,82	28,57
	Total	Pública	25	30,11	6,86	31,21	15,06	46,10	25	20,11	3,26	20,60	10,19	24,53
		Privada	25	35,46	7,38	35,51	18,76	48,85	25	25,10	5,84	25,08	13,20	39,30
5	Mono	Pública	25	43,71	6,03	43,90	34,78	54,55	25	35,35	8,61	36,14	12,50	55,56
		Privada	25	48,08	9,58	48,65	24,49	76,60	25	37,17	7,92	38,10	23,08	53,73
	Di	Pública	25	38,96	7,19	39,13	26,50	51,89	25	29,46	7,44	28,97	17,54	47,62
		Privada	25	41,04	6,05	41,94	29,80	49,23	25	33,84	6,40	33,77	17,54	44,78
	Tri	Pública	25	35,23	7,25	35,59	19,44	49,04	25	24,05	7,86	21,84	13,51	44,63
		Privada	25	36,98	7,23	36,84	22,27	48,09	25	25,12	5,19	25,79	14,71	35,92
	Poli4	Pública	25	28,71	7,81	29,90	15,57	43,64	25	16,57	4,59	15,58	10,99	27,66
		Privada	25	30,63	8,15	29,43	17,98	44,24	25	17,94	5,17	17,65	8,53	30,95
	Poli5	Pública	25	24,48	7,29	25,67	13,45	40,00	25	12,66	4,10	12,37	5,66	23,68
		Privada	25	27,62	7,10	27,27	16,22	39,34	25	14,20	4,70	12,77	6,11	23,41
	Total	Pública	25	32,63	6,67	33,13	21,92	44,10	25	21,31	4,55	20,49	15,25	31,86
		Privada	25	34,88	6,43	35,46	24,25	45,62	25	23,47	4,53	24,54	13,74	34,71

**Legenda:** DP: Desvio padrão; Mín.: Mínimo; Máx.: Máximo

## DISCUSSÃO

O objetivo do presente estudo foi dar seguimento ao processo de validação do Protocolo de Acompanhamento do Desenvolvimento da Decodificação (PRADE) em formato de *software* na etapa de evidência de validade baseada nos processos de resposta, tendo como participantes crianças de escola pública e privada.

Os dados trazem evidência de que o *software* utilizado mostra validade na análise dos processos de resposta, uma vez que foi possível caracterizar adequadamente o desempenho de crianças de escola pública e privada em todo o Ensino Fundamental I, tanto no que diz respeito a cada ano escolar quanto ao crescente desempenho positivo dos escolares em função do processo de escolarização. Além de caracterizar cada grupo, também mostra suas diferenças, que já são amplamente reconhecidas e discutidas em estudos anteriores, bem como em avaliações nacionais e internacionais^(5,6,11)^.

No que diz respeito ao tempo despendido para decodificar corretamente os estímulos, o fato de o primeiro ano de escola pública apresentar, por vezes, metade do número de crianças das escolas privadas indica que há um déficit importante na aprendizagem das relações grafofonêmicas, que podem decorrer de diversos cenários. É fundamental salientar que o PB é uma língua de média transparência, tendo maior transparência no sentido do grafema para o fonema do que o oposto^(21)^. Nesta perspectiva, alguns estudos mostraram que a demora para o domínio da habilidade de decodificação em idiomas mais transparentes pode estar relacionada à fatores extrínsecos à criança, como o método de ensino, escolaridade e práticas de literacia familiar, vulnerabilidade social e/ou déficits nas habilidades de alfabetização emergente^(2,11-14,17)^.

Assim sendo, é importante que se considere tais variáveis em estudos futuros para que o *software* caracterize e acompanhe a decodificação dos escolares, mas também considere os aspectos socioambientais dos indivíduos. Isso se torna fundamental pois uma meta-análise^(24)^ que objetivou verificar a correlação entre o desempenho em decodificação no início da alfabetização e o desenvolvimento posterior em leitura, mostrou evidências de que a decodificação é um forte preditor do desenvolvimento da leitura ao longo da vida acadêmica.

Dessa maneira, torna-se evidente a necessidade de acompanhamento precoce da evolução da criança em decodificação por meio de um instrumento rápido, democrático e em forma de *software* para amplo acesso como o PRADE. Desta forma, ter-se-á a possibilidade de desenvolver programas de formação de pais professores e estratégias coletivas para que, analisando-se a curva de resposta a tais atividades, possa-se atenuar a influência das variáveis extrínsecas e identificar rapidamente crianças com dificuldades intrínsecas e assim encaminhá-las para avaliação específica como consta nas diretrizes do DSM-5-TR^(25)^.

No que diz respeito à porcentagem de acertos e à acurácia de leitura, o mesmo fenômeno foi observado, desempenho superior de crianças de escola privada em ambos os estímulos, nas diferentes extensões e nos anos escolares. Importante ressaltar, entretanto, que tais diferenças tendem a diminuir a partir do terceiro ano escolar, conforme já apontado em estudos anteriores^(10,17,24,26)^ e também vão de encontro à Nova Política Nacional da Alfabetização^(27)^, que preconiza que os estudantes brasileiros devem dominar as habilidades básicas de decodificação até o final do segundo ano escolar. Alguns autores afirmam que o maior crescimento em medidas de leitura oral tem sido observado entre o primeiro e o terceiro ano de escolarização, ao contrário do que acontece entre o terceiro e o quinto ano, que tendem a apresentar menor variação nos resultados de avaliação^(10,12,17,26)^, dados corroborados neste estudo.

Cabe ressaltar que este padrão de desenvolvimento encontra substrato teórico na teoria de dupla rota (desenvolvimento da automaticidade da leitura). Dessa maneira, à medida que o indivíduo aprende a decodificar e dominar o código escrito, melhor é seu desempenho em testes de leitura, ou seja, à medida que seu léxico ortográfico aumenta, os valores encontrados na velocidade de decodificação tendem a se estabilizar e as diferenças tendem a ser menores com seus pares de escolaridade próxima^(1,10,13,17)^.

Os resultados aqui encontrados evidenciam curvas de desenvolvimento semelhante em todos os estudantes, com melhora em todas as variáveis em função do avanço da escolarização. Este dado confirma a hipótese da ciência cognitiva da leitura que ressalta a importância da aprendizagem e consolidação da decodificação nos primeiros anos escolares para se chegar à fluência e compreensão de leitura, objetivo final da aquisição de tal habilidade^(5,6,13,14)^. Cabe salientar, no entanto, a necessidade de investigação em diferentes tipos de escolas, de diferentes regiões do Brasil para que os dados sejam generalizados, sendo estas as próximas etapas do processo de validação do PRADE. É de fundamental importância que também se considere o desempenho de todos os escolares em função da extensão do estímulo, independentemente do tipo de escola, uma vez que se observou efeito contundente de tal variável em todos os anos escolares. Tais dados corroboram estudos anteriores^(11,26)^ que apontaram influência da extensão do estímulo no desempenho em decodificação de escolares brasileiros, evidenciando, mais uma vez, a efetividade do PRADE quanto à validade baseada nos processos de resposta.

Um estudo afirmou^(28)^ que as medidas de avaliação da decodificação, ao serem utilizadas como importantes marcadores e preditores do desempenho futuro em leitura, são de grande valia para pesquisadores, equipe escolar, profissionais que acompanham o desenvolvimento da leitura e seus transtornos e também para órgãos públicos de educação que monitoram o desempenho das crianças. Os pesquisadores realizaram uma meta-análise a fim de investigar se os valores considerados como corte para possíveis alterações no desenvolvimento de leitura tinham acurácia suficiente para realizar a devida aferição desta habilidade nas crianças. Os resultados das análises de periódicos, dissertações, teses e documentos públicos indicaram grande acurácia dos valores de corte dos diferentes estudos para a identificação de risco para a aquisição da leitura, reafirmando a avaliação da decodificação como uma importante ferramenta para identificação de possíveis atrasos no desenvolvimento da leitura^(4-6, 10-13)^.

Sendo assim, a literatura especializada na área bem como os resultados encontrados não apenas corroboram e sustentam os dados encontrados pelo PRADE, como também apontam para a necessidade de instrumentos de avaliação rápida, precisa e democrática da aquisição e desenvolvimento da decodificação desde os primeiros anos escolares, objetivo principal do PRADE.

## CONCLUSÃO

A etapa de evidência de validade baseada nos processos de resposta do *software* PRADE mostra-se adequada em termos de caracterização de grupos distintos da população-alvo do teste, bem como caracteriza adequadamente as crianças de escolas públicas e privadas de acordo com a faixa de escolaridade e também em relação a efeito da escolarização no processo de aquisição da decodificação de todos os estudantes. Assim sendo, o instrumento mostra-se assertivo e pronto para as próximas etapas de validação.
